# Levels of human platelet-derived soluble CD40 ligand depend on haplotypes of *CD40LG-CD40*-*ITGA2*

**DOI:** 10.1038/srep24715

**Published:** 2016-04-20

**Authors:** Chaker Aloui, Antoine Prigent, Sofiane Tariket, Caroline Sut, Jocelyne Fagan, Fabrice Cognasse, Tahar Chakroun, Olivier Garraud, Sandrine Laradi

**Affiliations:** 1 GIMAP-EA3064, University of Lyon, Saint-Etienne 42023, France; 2 French Blood Establishment, EFS Auvergne-Loire, Saint-Etienne 42023, France; 3 Regional Centre of Transfusion of Sousse, F. Hached University Hospital, Sousse 4000, Tunisia; 4 National Institut of Blood Transfusion (INTS), Paris 75015, France

## Abstract

Increased circulating soluble CD40 ligand (sCD40L) is commonly associated with inflammatory disorders. We aimed to investigate whether gene polymorphisms in *CD40LG, CD40* and *ITGA2* are associated with a propensity to secrete sCD40L; thus, we examined this issue at the level of human platelets, the principal source of sCD40L. We performed single polymorphism and haplotype analyses to test for the effect of twelve polymorphisms across the *CD40LG*, *CD40* and *ITGA2* genes in blood donors. *ITGA2* presented a positive association with rs1126643, with a significant modification in sCD40L secretion (carriers of C allele, P = 0.02), unlike the investigated *CD40LG* and *CD40* polymorphisms. One *CD40LG* haplotype (TGGC) showing rs975379 (C/T), rs3092952 (A/G), rs3092933 (A/G) and rs3092929 (A/C) was associated with increased sCD40L levels (1.906 μg/L (95% CI: 1.060 to 2.751); P = 0.000009). The sCD40L level was associated with the inter-chromosomal CD40LG/CD40/*ITGA2* haplotype (ATC), displaying rs3092952 (A/G), rs1883832 (C/T) and rs1126643 (C/T), with increased sCD40L levels (P = 0.0135). Our results help to decipher the genetic role of *CD40LG*, *CD40* and *ITGA2* with regard to sCD40L levels found in platelet components. Given the crucial role of sCD40L, this haplotype study in a transfusion model may be helpful to further determine the role of haplotypes in inflammatory clinical settings.

Clinical interest in CD40 ligand (CD40L, CD154) regulation is commonly reported in various inflammatory disorders^1^,^2^ and notably in relation to adverse events (AEs) after platelet transfusion. CD40L is mainly expressed on the surface of T cells, certain subsets of other leukocytes, endothelial cells and activated platelets^3^,^4^,^5^. CD40L binds to its preferred receptor CD40, thereby driving adaptive immune responses^6^.

Cell surface CD40L can be proteolytically cleaved by matrix metalloproteinases (MMPs) to generate soluble CD40L (sCD40L), which is biologically active as an important proinflammatory molecule and is also classified as a “Biological Response Modifier”^7^,^8^. Circulating sCD40L is known to be mainly derived from activated platelets *via* MMP-2^9^,^10^, which accounts for nearly 95% of the sCD40L in the plasma. sCD40L release increases in platelet components (PCs) under storage conditions and is directly responsible for febrile non-haemolytic transfusion reactions and other immediate transfusion adverse events (AEs)^11^,^12^,^13^,^14^,^15^.

Thus, we hypothesized the existence of a genetic risk factor in relation to the donor. In an initial study, we investigated the coding sequences, exon-intron junctions and regulatory regions of *CD40LG*, but we did not find any particular genetic pattern of *CD40LG* in two groups of individuals regardless of whether their donated platelets induced an AE^16^, despite the fact that two *CD40LG* polymorphisms are involved in *CD40LG* regulation, namely, sequence variations in the 5′ UTR of *CD40LG* (rs3092952)^1^ and a CA microsatellite in the 3′ UTR that affects mRNA stability^17^,^18^.

In the present study, we characterized the secretion of sCD40L in PCs destined for transfusion on day 0 of preparation (D_0_) and on the day of delivery (D_del_) in order to assess a possible genetic association between regulatory polymorphism and enhanced *in vitro* sCD40L release in PCs during storage. In most blood transfusion services, PC delivery is allowed from D_0_ to D_5_, with an average of D_4_ at worst and D_3_ at best. Moreover, sCD40L might also be modulated by independent genetic markers such as rs1883832 in the promoter region of the CD40 receptor^19^ and/or the C807T polymorphism (rs1126643) in the coding region of the platelet receptor for collagen (*ITGA2*). The latter was previously associated with some individual variation in platelet expression levels of GPIa/IIa (integrin α2β1), the preferential platelet receptor for collagen, which plays a crucial role in platelet adhesion and activation. This marker has further been defined as an independent predictor for the release of sCD40L^20^,^21^.

Therefore, the present study sought to highlight a possible genetic association (single markers and haplotypes) between 10 single nucleotide polymorphisms (SNPs) of *CD40LG*, rs1883832 of *CD40* and rs1126643 of *ITGA2*, which display enhanced *in vitro* sCD40L release in PCs during storage.

## Results

The genotype distribution for all investigated polymorphisms was found to be in Hardy-Weinberg equilibrium.

### Correlation between sCD40L levels and single polymorphisms

Relevant *CD40LG*, *CD40* and *ITGA2* polymorphisms were assessed.

No significant correlation was detected between the investigated *CD40LG* polymorphisms and sCD40L levels in the PCs, neither at D_0_ nor at D_del_, for all ten investigated CD40LG polymorphisms (Table 1).

There was also no association found between sCD40L levels and rs1883832 of the *CD40* gene, although this polymorphism has been shown elsewhere to regulate CD40L expression^19^,^22^.

However, there was a significant association with rs1126643 of *ITGA2*; the platelets of C-allele carriers (CC and CT) secreted elevated levels of sCD40L upon storage in shelf-life conditions–that is, with no deliberate stimulation–compared with the non-C carriers (TT homozygous), P = 0.08 at D_0_ and P = 0.02 at D_del_ (Table 1).

### Haplotype association with sCD40L levels

Five *CD40LG* haplotypes accounted for 97.6% of all potential combinations, including rs975379 (C/T), rs3092952 (A/G), rs3092933 (A/G) and rs3092929 (A/C). The association between *CD40LG* haplotypes and CD40L secretion leading to sCD40L is reported in Table 2. One haplotype (H_4_: TGGC; frequency: 2.6%) was associated with the largest increase in sCD40L levels at the day of PC delivery, i.e., 1.906 μg/L (95% CI: 1.060 to 2.751; P = 0.000009), compared with the reference haplotype H_1_ (CAGA). None of the other four CD40LG haplotypes was associated with any difference in sCD40L secretion and plasma levels.

Eight haplotypes accounted for 100% of potential multigene *CD40LG* (rs3092952)/*CD40* (rs1883832)/*ITGA2* (rs1126643) combinations. These haplotypes were tested for association with PC sCD40L levels. A significant association was shown for haplotype H_C_ at the time of preparation [H_C_: ATC; frequency: 10.8% with increased sCD40L level, i.e., 0.189 μg/L (95% CI: −0.182 to 0.560; P = 0.0135)], compared with the most common haplotype H_A_ (ACC; Table 3). It is interesting to note that haplotype H_G_ (GTC), which differs from H_C_ (ATC) by the first allele (A instead of G of the rs3092952 polymorphism), was also associated with a small non-significant increase in sCD40L levels at D_del_, i.e., 0.553 μg/L (95% CI: 0.235–0.87; P = 0.071). No other haplotype was consistently associated with sCD40L levels.

These results are reported after full adjustments for covariates, i.e., gender, age, number of platelets (10^9^/L) and the number of days of storage (inventory condition) prior to delivery. The major contributing factor exerting a positive effect on sCD40L levels was the platelet count (P = 0.000406 and P < 0.000001 at D_0_ and D_del_, respectively) for the *CD40LG* haplotype analysis. The same covariate was identified in the inter-chromosomal haplotype analysis (P = 0.001457 and P < 0.000001 at D_0_ and D_del_, respectively).

## Discussion

SNP association studies did not reveal any association with sCD40L levels measured in PCs at either D_0_ or D_del._ These results were consistent with our previous findings^16^ showing that no particular pattern of *CD40LG* in individuals who donated platelets by single apheresis and those in whom PCs induced an adverse transfusion reaction. It does seem, in light of the results and limitations of this study, that there are no so-called “regulator polymorphisms of *CD40LG*”, at least in healthy individuals.

Our findings did disagree with the observations of Malarstig *et al.*, who showed in a large cohort of patients with cardiovascular disease that carriers of the G allele of rs3092952^1^ had a 10% higher sCD40L level. However, to the best of our knowledge, this finding has yet to be consistently reproduced and despite this apparent correlation, rs3092952 did not confer an increased risk of cardiovascular adverse events. Regarding CA repeats in the 3′-UTR of *CD40LG*, our results corroborate those of Bugert *et al.*, indicating that neither the sizes of the alleles nor the genotypes of the CA repeat polymorphism were associated with plasma sCD40L levels^23^ (Table 1). Dai *et al.* found no association between CA repeats and mRNA expression in CD4 + T cells^24^. Only studies by Perez-Aciego *et al.* found that CD40L expression (membrane and mRNA) decreases in CD4 + T cells, but only in those with 24 CA alleles^25^,^26^. Taken together, these results indicate that the regulation of membrane CD40L expression and sCD40L levels is complex and implies not only genetic variations in *CD40LG*. Furthermore, one cannot exclude the possibility of environmental influences, cell-related post-translational regulation, catabolic regulation and/or polygenic control.

Extensive literature reported the association of the *CD40* -1C/T polymorphism (rs1883832) with CD40L expression. However, published results are difficult to compare because either the T or the C allele is implicated, with relatively equal frequencies between them^19^,^22^. The present study found no significant association with this polymorphism. Some of our unpublished data, however, confirm a positive correlation between surface protein CD40L expression and the rs1883832 genotypes present in the CD40 gene in T lymphocytes, in line with the findings of Zhang B *et al.*^22^.

The only genotype associated with sCD40L levels in PCs was found with C-allele carriers (CC and CT) of *ITGA2*, with a positive association (P = 0.02). This finding is discordant with the work of Antoniades *et al.*, who defined the T allele as an independent predictor for the release of sCD40L in healthy subjects but only in the subgroup with von Willebrand factor greater than or equal to the median^21^. Thus, again, an association with a specific allele (T or C) seems to be an ambiguous result, similar to other polymorphisms, e.g., *CD40*^19^,^22^. This polymorphism may affect either mRNA stability or a regulatory genetic region, with a subsequent change in the density of the expressed molecule on the platelet surface and, consequently, an alteration of platelet adhesion and activation, although this polymorphism does not alter the functional status of the protein^20^. This possibility may explain why the association between the *ITGA2* genotype and sCD40L levels was only found on the day of delivery, suggesting the requirement for sustained platelet activation over time, due to the preparation and storage process^27^,^28^.

We identified a highly significant association with the *CD40LG* haplotype at the day of PC delivery (H_4_: TGGC; P = 0.000009; Table 2). This result may be explained by progressive sCD40L release during platelet storage^13^,^27^. Moreover, we also identified one inter-chromosomal CD40LG/CD40/ *ITGA2* haplotype, H_C_ (ATC) from the rs3092952, rs18828232 and rs1126643 genotypes, associated with sCD40L levels (P = 0.0135; Table 3). This result highlights the importance of the association of several polymorphisms in different genes that are involved in the complex regulation of this immuno-modulatory molecule that is released after platelet activation. Notably, the frequency of H_C_ was nearly 11% of all the investigated *CD40LG/CD40*/ *ITGA2* haplotypes.

H_G_ was also associated with a relative increase in sCD40L levels at D_del_, although the difference did not reach statistical significance. This similarity could be linked to the similarity of the two haplotypes, H_G_ (GTC) and H_C_ (ATC), given that they show a difference in only the allele of the first rs3092952 polymorphism, which presents a regulatory function; however, that function was not identified in our study, which considered the polymorphism alone.

The apparent controversies between individual polymorphisms and haplotype analysis are explained by their different biological values and a higher informative analysis was attributed to haplotype investigation^29^. Sequential nucleotide variants may catch subtle changes in protein function, regardless of the presence of nucleic acid changes in the coding region^29^,^30^.

We identified two haplotypes associated with high levels of plasma sCD40L. However, a large fraction of CD40L is known to be carried by extracellular vesicles, including microvesicles and exosomes, after platelet activation^31^. As most methods used to assay sCD40L (i.e., ELISA and Luminex technologies) don’t distinguish between free sCD40L and microvesicle-carried-CD40L^32^,^33^, we believe that the levels of sCD40L measured in our study are not an underestimate.

To the best of our knowledge, this is the first study to suggest the haplotypic and polygenic control of sCD40L release from platelets stored for transfusion. This highly informative *in vitro* model contributes to a better understanding of the genetic role of *CD40LG*, *CD40* and *ITGA2* with regard to sCD40L platelet secretion and the subsequent levels in PCs and, consequently, the role of sCD40L in AEs. However, we cannot exclude that the biological response may also be affected by pathophysiologic aspects and co-morbidities in the transfusion recipients. The replication of the associations we found, as well as additional functional studies in pathological situations with inflammatory disorders, is required to confirm our findings in a larger number of individuals and must be extended to other clinical settings (e.g., *in vivo* models).

## Methods

### Subjects

#### Ethics statement

The study was carried out in accordance with the Helsinki Declaration and approved by the ethical committee of the F. Hached University Hospital, Sousse, Tunisia. Informed and written consent was obtained from all the healthy donors who participated in this study.

#### Study population

The studied cohort comprised 142 volunteer blood donors, including 52 males and 90 females, 26 ± 10 years of age (mean ± SD), who donated whole blood at the Transfusion Centre of Sousse. Individual PCs were derived from each donation as described^34^. None of the blood donors were family-related; donors entered the study randomly, on the sole basis of the timing of their donations; no selection criteria specific to this study were applied.

### Genotyping

Genomic DNA was obtained from peripheral venous blood using the FlexiGene DNA Kit (Qiagen, Paris, France). For *CD40LG*, rs3092952 A/G and CA repeats were genotyped as previously described using denaturing High Performance Liquid Chromatography (dHPLC) and capillary electrophoresis, respectively^16^. The remaining 8 *CD40LG* polymorphisms were genotyped via multiplex PCR amplification in two groups of quadruplex Tetra primer Amplification Refractory Mutation System-PCR (T-ARMS-PCR) followed by fragment analysis (the first group included rs3092945 C/T, rs975379 C/T, rs3092929 A/C and rs3092920 G/T; the second group included rs3092948 C/G, rs3092927 A/G, rs715762 C/T and rs3092933 A/G)^35^,^36^,^37^,^38^,^39^. Each of the two groups of primers used for the amplification of twelve fragments of different sizes was pooled in a single 25-μL reaction volume, which contained 12.5 μL of master mix from the Multiple × PCR Kit (Qiagen, Paris, France), according to the manufacturer’s recommendations. PCR conditions included an initial denaturation step of 95 °C for 10 min, followed by 7 cycles of 94 °C for 30 s, 70 °C minus 1 °C per cycle for 30 s and 72 °C for 30 s. Then, we performed 29 cycles of 94 °C for 30 s, followed by annealing for 30 s at 65 °C and 63 °C for multiplex T-ARMS-PCR groups I and II, respectively^40^.

Genotype data were integrated with two additional SNPs, mapping at *CD40 (*rs1883832 C/T) and at *ITGA2* (rs1126643 C/T), genotyped by single T-ARMS-PCR under the same technical conditions, with annealing temperatures of 67 °C and 67.5 °C for *CD40* and *ITGA2*, respectively (Supplementary Tables S1 and S2).

### Soluble CD40L assay

Non-leukodepleted, individual PCs were prepared according to the standard protocol of the blood bank of Sousse^34^. Under sterile conditions, 4 mL was derived from PCs, first on the day of blood donation and platelet bag preparation (D_0_) and again on the day of delivery (D_del_). The samples were centrifuged at 180 g for 10 min and supernatants were collected and frozen at −80 °C and then thawed immediately before being assayed at room temperature. Soluble CD40L in PC supernatants was measured using a commercially available method by Luminex Technology according to the manufacturer’s instructions (Milliplex Map Kit Millipore, Darmstadt, Germany).

### Statistical analysis

All statistical analyses were performed with XLSTAT^TM^ software (Addinsoft, Paris, France) using non-parametric methods. The Hardy-Weinberg equilibrium test was used to control the genotyping results. We used the Kruskal-Wallis test to evaluate the association between the genetic polymorphisms and sCD40L levels.

To test for the prevalence of genotypes of the CA repeat polymorphism, we defined three types as referred to Table 1 as follows: (i) genotype ‘only 26′ with the 26 CA allele exclusively (this group comprised females with two 26 alleles and males with one 26 allele, because the gene is X-linked); (ii) genotype ‘26+’ with any allele other than 26 in addition to one 26 allele (heterozygous females); and (iii) genotype ‘no 26’ with no 26 allele (any genotype without the 26 allele).

**Figure 1:**
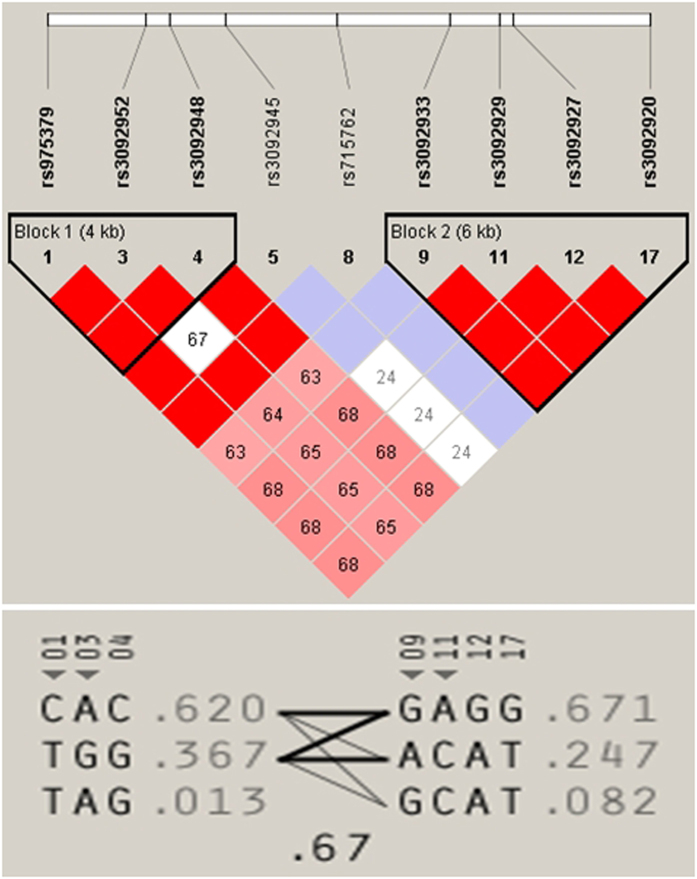
Representative LD plot of the two haplotype blocks at *CD40LG* and the tagged SNPs used for the haplotype association.

For haplotype association, we used Haploview 4.2 software and employed the algorithm proposed by Gabriel^41^ in order to choose tagged SNPs based on our previous study^16^. The haplotype tagger function was used to identify redundant SNPs, which were considered redundant if the pairwise LD (r^2^) was ≥0.8. Haplotypes with a frequency ≤1% were excluded from the analysis (Fig. 1).

The consequences of displaying haplotypes on the propensity for platelet secretion of sCD40L were evaluated using THESIAS v3.1 software^42^. The association of each haplotype with sCD40L levels was measured using a regression parameter and the 95% confidence interval (CI), where the effect of each haplotype is compared with the most frequent haplotype, termed the ‘reference’ in the regression model. Adjustments were performed for different covariates, such as gender, age, platelet count and storage length prior to delivery (considered when testing for sCD40L at D_del_).

All significance thresholds were set at P < 0.05.

## Additional Information

**How to cite this article**: Aloui, C. *et al.* Levels of human platelet-derived soluble CD40 ligand depend on haplotypes of *CD40LG-CD40-ITGA2. Sci. Rep.* **6**, 24715; doi: 10.1038/srep24715 (2016).

## Supplementary Material

Supplementary Information

## Figures and Tables

**Table 1:** Frequency distribution of *CD40LG*, *CD40* and *ITGA2* genotypes, considering sCD40L level, on the day of preparation (D_0_) and on the day of PC delivery (D_del_).

Gene	Polymorphism	Genotype	N	D_0_: Median (1^st^–3^rd^Quartile)	p-value	D_del_: Median (1^st^–3^rd^ Quartile)	p-value
*CD40LG*	rs3092952	AA+A	69	560.34 (336.82–916.4)	0. 99	854. 78 (534. 26–1148. 66)	0. 33
		AG	43	496.44 (303.98–967.47)		781. 84 (472. 23–1266. 56)	
		GG+G	30	589.45 (362.42–791.13)		1121. 455 (564. 897–1353. 59)	
	CACA	26+/other	37	498.36 (336.82–1123.88)	0. 55	746. 72 (431–1266. 56)	0. 3
		Only 26	24	444.4 (230.86–898.925)		682. 74 (515. 15–1253. 45)	
		No 26	81	556 (361.88–908.76)		924. 54 (584. 18–1251. 6)	
	rs975379	CC+C	70	547.5 (340.265–914.49)	1	853. 91 (535. 15–1144. 325	0. 35
		CT	42	502.08 (301.73–973.085)		799. 8 (471. 455–1266. 56)	
		TT+T	30	589.45 (362.42–791.13)		1121. 455 (564. 898–1353. 59)	
	rs3092948	CC+C	66	589.93 (340.265–920.075)	0. 9	865. 64 (526. 025–1187. 405)	0. 47
		CG	44	494.97 (306.23–961.855)		799. 8 (473. 005–1266. 56)	
		GG+G	32	527.18 (360.925–765.35)		1084. 295 (573. 613–1347. 33)	
	rs3092945	CC+C	6	875.95 (531.935–1022.295)	0. 31	1186. 315 (694. 273–1256. 79)	0. 25
		TC	22	364.16 (272.27–834.385)		678. 66 (471. 455–1079. 625)	
		TT+T	114	581.25 (358.115–914.49)		900. 52 (542. 5–1340. 11)	
	rs715762	CC+C	127	546.48 (345.99–924.24)	0. 6	853. 04 (517. 89–1255. 06)	0. 32
		CT	8	370.36 (271.215–706.9)		851. 46 (515. 415–1158. 05)	
		TT+T	7	642.6 (430.32–930.08)		1199. 83 (940. 05–1350. 46)	
	rs3092933	AA+A	14	693.37 (391.3–791.13)	0. 9	944. 07 (516. 2–1226. 195)	0. 56
		GA	37	546.48 (362.84–927.18)		932. 62 (560. 54–1594. 4)	
		GG+G	91	498.36 (295.56–949.43)		839. 96 (536. 1–1200. 075)	
	rs3092929	AA+A	80	495.34 (284.07–898.925)	0. 34	847. 37 (574. 81–1234. 04)	0. 55
		AC	38	494.97 (359.945–986.99)		835. 4 (452. 68–1256. 1)	
		CC+C	24	693.37 (408.945–918.54)		1037. 81 (571. 483–1275. 85)	
	rs3092927	AA+A	22	693.37 (419.265–892.89)	0. 3	1084. 295 (558. 508–1310. 51)	0. 58
		GA	39	496.44 (372.19–987.97)		853. 04 (456. 06–1245. 64)	
		GG+G	81	492.32 (288.94–893.1)		839. 96 (556–1223. 2)	
	rs3092920	GG+G	77	498.36 (288.94–893.1)	0. 44	876. 5 (556–1266. 56)	0. 83
		GT	39	546.48 (381.58–1060.56)		818. 48 (453. 36–1245. 64)	
		TT+T	26	632.57 (382.26–834.965)		980. 34 (516. 2–1256. 79)	
*CD40*	rs1883832	CC	82	553.41 (338.47–910.575)	0. 93	895. 93 (591. 49–1244. 88)	0. 62
		CT	52	531.86 (312.11–922.77)		865. 64 (467. 36–1271. 075)	
		TT	8	451.22 (377.53–1087.42)		544. 175 (386. 4–1472. 04)	
*ITGA2*	rs1126643	CC	53	534.66 (299.48–868.98)	0. 08	876. 5 (490. 7–1224. 72)	0. 02
		CT	64	647.6 (361.455–1009.2)		998. 95 (639. 67–1621. 995)	
		TT	25	401.28 (218.04–657.18)		691. 5 (454. 72–942. 56)	

The association analysis between sCD40L level and rs1126643 in ITGA2 on the day of delivery is detailed as follows: CC vs CT = 0.095; CC vs TT = 0.167; TT vs CT = 0.006.

**Table 2:** Frequency distribution of *CD40LG* haplotypes in PCs and their interaction with sCD40L level, considering sCD40L level, on the day of preparation (D_0_) and on the day of PC delivery (D_del_).

*CD40LG* haplotype (rs975379, rs3092952, rs3092933, rs3092929)			Haplotype interaction with sCD40L			
	Haplotype	Frequency (N = 142)	sCD40L mean (95% CI) at D_0_	P-value	sCD40L mean (95% CI) at D_del_	P-value
H_1_	CAGA	0.578	−0.122 (−0.776−0.531)	reference	0.470 (−0.098–1.040)	reference
H_2_	CAGC	0.057	0.172 (−0.922–0.127)	0.484	0.203 (−0.978–1.384)	0.594
H_3_	TGGA	0.105	−0.037 (−0.690–0.615)	0.698	0.353 (−0.336–1.043)	0.639
H_4_	**TGGC**	**0.026**	−0743 (−1.357 −1.208)	0.927	**1.906 (1.060–2.751)**	0.000009*
H_5_	TGAC	0.210	−0.123 (−0.829–0.583)	0.997	0.509 (−0,272–1.291)	0.843
Other		0.024				

Data represent the mean and relative 95% CI of the difference in platelet supernatant sCD40L levels (μg/L) observed in one copy of each haplotype configuration compared with the reference haplotype. Haplotypes (Hn) are indicated in ACGT format with rs975379 (C/T), rs3092952 (A/G), rs3092933 (A/G) and rs3092929 (A/C). The data are boldfaced if the corresponding haplotypes show a significant P-value (<0.05). All data were adjusted for gender, age and platelet count. Data on D_del_ were further adjusted for the number of days before delivery.

**Table 3:** Frequency distribution of *CD40LG/CD40/ ITGA2* haplotypes in PCs and their interaction with sCD40L levels.

*CD40LG* (rs3092952)/ *CD40* (rs1883832)/ *ITGA2* (rs1126643)			Haplotype interaction with sCD40L			
	Haplotype	Frequency (N = 142)	sCD40L mean (95% CI) at D_0_	P-value	sCD40L mean (95% CI) at D_del_	P-value
H_A_	ACC	0.277	−0.118 (−0.461–0.226)	reference	0.248 (−0.345–0.531)	reference
H_B_	ACT	0.226	−0.097 (−0.423–0.228)	0.869	0.293 (−0.444–0.631)	0.768
**H_C_**	**ATC**	**0.108**	**0.189 (−0.182–0.560)**	0.0135*	0.242 (−0.195–0.678)	0.975
H_D_	ATT	0.033	−0.201 (−1.083–0.681)	0.857	0.301 (0.188–0.789)	0.827
H_E_	GCC	0.161	−0.084 (−0.398 −0.231)	0.828	0.390 (0.009 −0.771)	0.350
H_F_	GCT	0.093	−0.002 (−0.343–0.340)	0.394	0.145 (−0.426–0.717)	0.715
H_G_	GTC	0.058	−0.141 (−0.820–0.539)	0.936	0.553 (0.235–0.872)	0.071
H_H_	GTT	0.044	−0.201 (−0.108–0.681)	0.993	0.130 (−0,694–0.954)	0.754

Data represent the mean and relative 95% CI of the difference in platelet supernatant sCD40L levels (μg/L) observed in one copy of each haplotype configuration compared with the reference haplotype. Haplotypes (Hn) are indicated in ACGT format with rs3092952 (A/G), rs1883832 (C/T) and rs1126643 (C/T). The data are boldfaced if the corresponding haplotypes show significant P-values (<0.05). All data were adjusted for gender, age and platelet count. Data on D_del_ were further adjusted for number of days before delivery.
