# Supplementary material for: Levels of human platelet-derived soluble CD40 ligand depend on haplotypes of CD40LG-CD40-ITGA2
Source: Sci Rep. 2016 Apr 20;6:24715. doi: 10.1038/srep24715 (PMC4837387; doi:10.1038/srep24715)
Supplement: Supplementary Information [file srep24715-s1.doc]

**Levels of human platelet-derived soluble CD40 ligand depend on haplotypes of *CD40LG-CD40*-*ITGA2***

Chaker Aloui^1,2^, Antoine Prigent^1,2^, Sofiane Tariket^1^, Caroline Sut^1^, Jocelyne Fagan^2^, Fabrice Cognasse^1,2^, Tahar Chakroun^4^, Olivier Garraud^1,5^, Sandrine Laradi^1,2^.

**Supplementary Table S1.** CD40LG primers for the 2 quadruplex T-ARMS-PCR groups.

**Supplementary Table S2.** CD40G *and ITGA2* primer sequences for T-ARMS-PCR

**Table S1.** *CD40LG* primers for the 2 quadruplex T-ARMS-PCR groups

|  | **SNP** | **Primer** | **Sequence^*^ (5’->3’)** | **Tm (°C)** | **C**† **(µM)** | **Length (bp)** | |
| --- | --- | --- | --- | --- | --- | --- | --- |
|  |  |  |  |  |  | **Specific bands** | **Control bands** |
| Group I | **rs975379**  C>T | F1 rs379 | TTCTTCCAGAATTCCCTTCAAAGCATG***C***C | 72.7 | 0.2 | 145 | 519 |
|  |  | R1 rs379 | CCAGGTCCCCAAGGTGAGCTTTCTTG | 72.6 | 0.12 |  |  |
|  |  | F2 rs379 | CACTTCTCTACTGTGGATGCTTACCCAGTGC | 72 | 0.08 | 426 |  |
|  |  | R2 rs379 | CCCACCCCAAACACAGCAGGAGAA | 72.7 | 0.12 |  |  |
|  | **rs3092945** T>C | F3 rs945 | CCTATTTTCCCTATTCTGAACTGTTACATCAGCAT | 69.1 | 0.2 | 125 | 258 |
|  |  | R3 rs945 | AGACTCTGGAATTGAAGTTAAAGTAAAAATCGTGAC | 68.1 | 0.14 |  |  |
|  |  | F4 rs945 | ATAAGCAAGACAGGTGCAAGTGCCTCCT | 70.9 | 0.1 | 202 |  |
|  |  | R4 rs945 | GTGTACACTGTTCCAATCCATTAGATAATTGTT***A***G | 65.9 | 0.2 |  |  |
|  | **rs3092929** A>C | F5 rs929 | ACTGGGAGCATCCCTCCTCCTAACC***T***A | 70 | 0.1 | 174 | 349 |
|  |  | R5 rs929 | TGTAACGTCTTGGGACCTATTGATCTTTACAG | 68.4 | 0.1 |  |  |
|  |  | F6 rs929 | TCTTCAAAGTGAGTTCAAATGCACAGATGGG | 72.5 | 0.1 | 230 |  |
|  |  | R6 rs929 | CATGAGGCCCATTTCCACACAGTGAAG***C***G | 77.8 | 0.1 |  |  |
|  | **rs3092920** G>T | F7 rs920 | GCAAAAATTTGCTACACTGAAAGGATCCTCATTAG | 70.8 | 0.2 | 467 | 704 |
|  |  | R7 rs920 | GCACCCCTGTTCTGACAGCTTGCTAGAG | 72.5 | 0.2 |  |  |
|  |  | F8 rs920 | GCACCCTGCACTTAGCAAGTGCTTCACA | 74.3 | 0.1 | 306 |  |
|  |  | R8 rs920 | ATGAGTGCATTTTTATCATCCAGATATTTTTGACA | 69.2 | 0.1 |  |  |
| Group II | **rs3092948** C>G | F1 rs948 | CAGAAGGATGAGAGAAAGAGAAGGCTTCAAC | 69.2 | 0.2 | 158 | 290 |
|  |  | R1 rs948 | CCTCTCCTTTGTAGATTACTGAATTGTACCATCCT | 68.8 | 0.1 |  |  |
|  |  | F2 rs948 | CCTGTTAGGGAGAGACAGGCATGAGAGG | 71.2 | 0.1 | 189 |  |
|  |  | R2 rs498 | CAGAATGCCCTGCTTGTACCTCAATTC | 69.2 | 0.1 |  |  |
|  | **rs715762** C>T | F3 rs762 | AGAACACTGAGTCAAATACCCTTGGGCC | 70.5 | 0.1 | 320 | 479 |
|  |  | R3 rs762 | AGAACCTGAAAGCAAGCAGCGGATTT | 70 | 0.1 |  |  |
|  |  | F4 rs762 | GCTAGCATGAAGTCACTGCAGTGACTGTG | 71.3 | 0.1 | 214 |  |
|  |  | R4 rs762 | CCTAGAAATGACCCCTAAGTGAGCCG***C***A | 72.4 | 0.14 |  |  |
|  | **rs3092933** G>A | F5 rs933 | GGCAGCCTGATCCGTCTTTGAAT***T***G | 71.1 | 0.1 | 265 | 438 |
|  |  | R5 rs933 | AACAGTGGAAGACATCAGGGATCTTTAGCC | 70.1 | 0.1 |  |  |
|  |  | F6 rs933 | TTATTTGCCCGGTTCTTAAAGTGAGAGCAT | 69.7 | 0.3 | 239 |  |
|  |  | R6 rs933 | GACTTCTCTGGAGTAGTATAGTAATCTTGGTATTATTAGA***A***T | 63.9 | 0.3 |  |  |
|  | **rs3092927** G>A | F7 rs927 | TGTGTGTGCATATGTGTATGTGTGTGACA***T***G | 72.3 | 0.08 | 404 | 714 |
|  |  | R7 rs927 | GGGGCTTTATGTCACCCTTTTGACATCTAA | 70.3 | 0.12 |  |  |
|  |  | F8 rs927 | TCTGTAAAGATCAATAGGTCCCAAGACGTTACA | 69 | 0.2 | 374 |  |
|  |  | R8 rs927 | TTTTTTCTCTCTCTGTCCATCTCTCTTTCTC***G***CT | 71 | 0.2 |  |  |

* Specific nucleotides are underlined; mismatches are presented in bold and italics

† Final reaction concentration

The specificity of the T-ARMS-PCR specific primer sequences was enhanced by introducing an additional mismatch at the penultimate or third nucleotide from the 3' end of the primer when necessary.

**Table S2.** *CD40G* *and ITGA2* primer sequences for T-ARMS-PCR

|  | **SNP** | **Primer** | **Sequence^*^ (5’->3’)** | **Tm (°C)** | **C**† **(µM)** | **Length (bp)** | |
| --- | --- | --- | --- | --- | --- | --- | --- |
|  |  |  |  |  |  | **Specific bands** | **Control bands** |
| *CD40* | **rs1883832** C>T | F1 CD40 | GCCGCCTGGTCTCACCTCGCT | 72.7 | 0.20 | 263 | 573 |
|  |  | R1 CD40 | ACCCCAGCCCGGGAGAAGAGAGA | 72.4 | 0.11 |  |  |
|  |  | F2 CD40 | GGTCGCAGGAGCAGGCTAGCTCC | 71.5 | 0.20 | 355 |  |
|  |  | R2 CD40 | GCACTGCAGAGGCAGACGAACCGTG | 75.9 | 0.20 |  |  |
| *ITGA2* | **rs1126643** C>T | F1 ITGA2 | AATATGGTGGGGACCTCACAAACACATTC | 70.9 | 0.22 | 235 | 459 |
|  |  | R1 ITGA2 | CCCAGCTGCCTTCTCAAAGTATTCAAGAC | 70.3 | 0.42 |  |  |
|  |  | F2 ITGA2 | GGAAGTGATGCCTTAAAGCTACCGGC | 69 | 0.47 | 284 |  |
|  |  | R2 ITGA2 | CCAAAACTTACCTTGCATATTGAATTGCTCCA | 71 | 0.28 |  |  |

* Specific nucleotides are underlined; mismatches are presented in bold and italics

† Final reaction concentration

The specificity of the T-ARMS-PCR specific primer sequences was enhanced by introducing an additional mismatch at the penultimate or third nucleotide from the 3' end of the primer when necessary.
